# Effect of Prolonged Use of Different Facemasks on Their Physical Performance and Physiological Impact on the Wearer

**DOI:** 10.1016/j.shaw.2025.06.001

**Published:** 2025-06-30

**Authors:** Baderin Osman, Muhammad Zubir Yusof, Haalah Mahmud

**Affiliations:** 1 Consultation Research and Development Department, National Institute of Occupational Safety and Health, Bandar Baru Bangi, Selangor, Malaysia; 2 Department of Community Medicine, Kulliyyah of Medicine, International Islamic University Malaysia, Kuantan, Pahang, Malaysia

**Keywords:** breathing resistance, facemask, facial skin temperature, penetration, transepidermal water loss

## Abstract

**Background:**

Most published studies on potential facemask physical performances and physiological impairments had shorter observation periods, and the observed occupational physical activities had limited relevance to real occupational work. Thus, our study aimed to assess the impact of prolonged mask use on its physical performances and the associated physiological responses in wearers.

**Methods:**

The recruitment of study participants took place between November 2022 and March 2023. Facemask penetration and breathing resistance measurements were obtained at 4 and 8 hours using the TSI Model 8130 Automated Filter Tester and the INSPEC Breathing Resistance Rig. Facial skin temperature and transepidermal water loss were quantified using thermal imaging cameras and vapometers, respectively.

**Results:**

A total of 216 workers participated in the study. A significant reduction in penetration (p < 0.001, partial Ƞ^2^ = 0.1) and an increase in breathing resistance (p < 0.001, partial Ƞ^2^ = 0.9) were observed only in case of surgical masks worn by cleaners after 8 hours. Facial skin temperature increased after 8 hours for KF94 (p < 0.001, partial Ƞ^2^ = 0.2), surgical mask (p < 0.001, partial Ƞ^2^ = 0.4), and cloth mask (p < 0.001, partial Ƞ^2^ = 0.2). All three facemasks had a statistically significant interaction with use on facial skin temperature. Higher transepidermal water loss was only observed for the cloth mask (F (p = 0.034, partial Ƞ^2^ = 0.02).

**Conclusion:**

Our findings suggest that prolonged use of face masks can lead to a deterioration in penetration, breathing resistance, and physiological impairment for the mask wearer. The implications are particularly critical for high–occupational activity jobs requiring prolonged use of masks.

## 1 Introduction

The widespread usage of face masks has become an essential public health strategy in reducing the transmission of respiratory illnesses, especially during the COVID-19 pandemic. Although face masks have been extensively shown to be effective in lowering transmission, there is increasing concern about the possible consequences of prolonged mask usage on both the mask's physical functionality and the wearer's well-being. In general, a facemask should only be worn for a limited period of time, but due to personal protective equipment shortages, prolonged usage of a facemask happened during the COVID-19 pandemic [1]. Despite the implementation of guidelines for the prolonged use, reuse, and reprocessing of single-use masks and respirators, there is still no consensus on the exact duration of facemask usage [2].

Several factors, including the type of mask, material composition, and duration of wear, influence the physical performance of face masks. Studies have shown that prolonged use can lead to mask deformation, reduced breathability, and a decline in filtration efficiency [3]. These changes may not only decrease the protective capacity of the mask but also increase the wearer's exposure to airborne particles, thereby undermining the intended purpose of the mask.

Additionally, prolonged use of face masks has been associated with various physiological effects on the wearer. Studies have shown that prolonged mask use, especially with N95 respirators and surgical masks, significantly affects physiological responses and increases discomfort [4]. Long-term use can alter gas exchange, while even short-term use during physical activity can impact heart rate, oxygen and carbon dioxide levels, respiratory rate, and facial skin temperature [5,6]. The accumulation of heat and moisture inside the mask creates an uncomfortable microclimate [7], leading to skin issues such as itching, rashes, redness, and pressure bruises [[8], [9], [10]]. Continuous mask use for more than 12 hours has resulted in nasal bridge scarring and facial itching, while mask use for more than four hours a day has been associated with headaches and other respiratory challenges due to carbon dioxide buildup [[11], [12], [13]].

Three types of facemasks have been widely utilized during the COVID-19 pandemic: cloth masks, surgical masks, and filtering facepiece respirators, which include KF94. Cloth masks, typically made from materials like cotton or fabric, are homemade and provide limited filtration efficiency, mainly recommended for community transmission. Their thick fibers and large pores hinder fine particle filtration, and efforts to improve performance by adding layers or altering fiber structure increase breathing resistance and discomfort, making prolonged wear difficult [[14], [15], [16]]. Surgical masks, designed primarily to reduce bacterial spread rather than protect against airborne particles, offer better filtration than cloth masks due to their three-layer structure (spunbonded outer and inner layers and a melt-blown filter layer), which features smaller pores and a denser fabric [[17], [18], [19]]. Facepiece respirators like N95s, which offer 95% filtration efficiency, provide superior protection. KF94, a Korean equivalent, offers 94% filtration. Although N95s provide better protection, KF94 and KN95 masks may be more accessible in certain regions. KF94 masks consist of multiple layers, including nonwoven polypropylene fabric in the inner and outer layers, and a polypropylene filter layer [20].

In Malaysia, the mandatory and widespread use of face masks has been a key measure in controlling infectious diseases, particularly during the COVID-19 pandemic. Given their importance in infection prevention, it is crucial to understand how prolonged mask use affects both mask performance and the wearer's physiological health. Authorities should consider potential health risks when making workplace mask-wearing recommendations. Therefore, further research is necessary, focusing on longer wearing periods and work-related scenarios. Thus, this study aims to assess the effects of prolonged use of KF94, surgical, and cloth masks on physical performance and physiological responses including particle penetration, breathing resistance, facial skin temperature, and transepidermal water loss (TEWL) in the context occupational physical activity (OPA).

## 2 Methods

### 2.1 Study recruitment and data collection

A cross-sectional study was conducted from November 2022 to March 2023, recruiting 240 workers from the National Institute of Occupational Safety and Health (NIOSH) headquarters in Malaysia using universal sampling. Before completing the questionnaire and data collection, participants were given a brief overview of the study's objectives and assured of anonymity and confidentiality. Participation was voluntary, with no personal information required, and participants could withdraw at any time. No incentives were offered for participation, and all participants signed a consent form. Workers were categorized into three job roles based on their OPA: cleaners (high OPA), trainers (moderate OPA), and office workers (low OPA) [21]. Cleaners engaged in physically demanding tasks such as sweeping, mopping, lifting, and waste handling. Trainers were involved in teaching and demonstrating safety procedures, which required intermittent physical activity. Office workers (low OPA) primarily conducted sedentary tasks, including desk work, documentation, and attending meetings.

Participants were recruited based on specific inclusion and exclusion criteria. Individuals were eligible to participate if they met the following criteria: (i) provided written informed consent; (ii) were able to understand either Malay or English; (iii) were willing to wear three types of face masks (KF94, surgical, and cloth) across two durations (4 hours and 8 hours) over six consecutive days; and (iv) agreed to undergo both baseline and post–mask-wearing physiological measurements, including facial skin temperature and TEWL. Participants were excluded if they self-reported any pre-existing medical condition, such as symptoms consistent with COVID-19 infection or any respiratory disease that could interfere with their ability to wear a face mask.

All participants were instructed to wear three different types of face masks: KF94, surgical, and cloth, across two durations: 4 hours and 8 hours. Each participant wore one type of mask per duration each day, rotating through all three mask types over six consecutive days. Mask-wear durations were set at 4 and 8 hours to represent short-to-moderate and prolonged use, respectively. The 8-hour duration reflects a typical full work shift and is supported by previous research indicating that mask protection remains stable up to 8 hours, after which wearer health risks may increase [22].

Prior to the study, participants were provided with masks and given instructions on the procedures for each sampling day. Baseline skin physiology measurements, including skin temperature and TEWL, were taken without a mask. Participants then wore the assigned mask for the specified duration while continuing their regular daily occupational activities, defined as routine job tasks. Postwear measurements were collected at the end of the assigned period, after which participants removed the mask and placed it in sterilized zip-lock bags for further analysis. Each mask was labeled with the participant's identification to ensure proper tracking for subsequent physical performance evaluation.

### 2.2 Study instrument

#### 2.2.1 Background characteristics

The self-administered questionnaire was used to collect background information about the respondents, including their sociodemographic status, socioeconomic status, and job descriptions such as age, gender, level of education, household income, smoking status, and type of occupation.

#### 2.2.2 Facemask physical performance

All masks were evaluated for particle penetration and breathing resistance in accordance with the Malaysian Standard (MS2323:2010) for testing procedures, requirements, and markings [23]. A TSI Model 8130 Automated Filter Tester (TSI, Inc., St. Paul, MN) was used to measure aerosol penetration. This device evaluates particulate respirator filters and filter media using sodium chloride (NaCl) aerosol with a count median diameter of 0.06–0.16 μm, a mass median diameter of 0.18–0.4 μm, and a geometric standard deviation of approximately 1.9–2. The instrument features an accuracy of ±2% of full scale for both aerosol flow and pressure measurements. Particle penetration was calculated as the ratio of upstream to downstream aerosol concentrations, simultaneously measured by dual-light-scattering laser photometers, enabling a fully automated and precise assessment. The airflow of the NaCl aerosol was set at approximately 28.3 liters per minute (L/min). The effectiveness of each mask configuration in blocking aerosols, along with the averages, was reported [24]. The breathing resistance was tested in accordance with AFNOR SPEC S76-001 standards [25] using the INSPEC Breathing Resistance Rig at a constant flow rate of 160 L/min. The inhalation reading was recorded in millimeters of water (mmH_2_O), with the equipment having an accuracy of ±0.2% of the reading, ±0.2% of full scale, and ±1 digit**.**

#### 2.2.3 Physiological effects

Facial skin temperature measurements were taken on the mask-covering area including the cheeks, perioral area, and chin [9] using a Chauvin Arnoux DiaCAM 2 thermal imaging camera, with the data analyzed using CAmReport® software. This device operates within a temperature range of –20°C to +250°C with an accuracy of ±2°C or ±2% of the reading. To prevent exposure to intense thermal radiation, measurements were conducted indoors in an enclosed office setting. In addition, a vapometer (Delfin Technology Ltd, Kuopio, Finland) was utilized in this study to assess TEWL in the same facial areas. The instrument provides rapid measurements (within 10–20 seconds) and operates accurately up to 300 g/m^2^/h. Its closed-chamber diffusion technology minimizes environmental interference, making it suitable for use outside controlled laboratory settings. The device's design allows for flexible, orientation-independent measurements and does not require calibration, ensuring consistent and user-friendly operation during field assessments. The flowchart of the TEWL measurement process is provided in the Appendix A.

### 2.3 Data analysis

All the collected data were analyzed using the IBM Statistical Package for the Social Sciences version 24.0 (SPSS Statistics 2.0) [26]. Descriptive statistics were used to analyze background characteristics, physical performances of facemask, and physiological effects of the facemask wearer based on type of mask and occupation. A mixed-repeated-measure analysis of variance was conducted to compare physical performance measures and physiological effects of the facemask wearer across the two time periods (4 hours and 8 hours of facemask wearing), with facemask type as a between-subjects factor. A *p* value <0.05 was considered statistically significant.

### 2.4 Ethical consideration

This study was approved by the NIOSH Ethics Committee (JPE) (NIOSH/03/JEP/2022(21)).

## 3 Results

### 3.1 Background characteristics

A total of 216 respondents participated in the survey, with a 90% response rate. More than half of the workers were male (56.5%) and under 40 years of age (55%). In terms of occupation, a high proportion of males were trainers (88.2%), with 52.9% of them being 40 years or older. The majority of workers had a high level of education (94.9%), and 73.1% of them had a household income in at least the M40 category group (earned less than United States Dollar [USD] $1721 per month). All the cleaners were in the B40 household income group (earned less than USD $769 per month). A significant proportion of the workers were nonsmokers (75%), regardless of occupation (Table 1).

**Table 1:** Background characteristics (*N* = 216)∗

Characteristics	Overall		Type of occupation					
			Trainer		Office worker		Cleaner	
	*N*	%	*N*	%	*N*	%	*N*	%
Age								
<40 years old	119	55.1	24	47.1	86	55.8	9	81.8
≥40 years old	97	44.9	27	52.9	68	44.2	2	18.2
Gender								
Male	122	56.5	45	88.2	75	48.7	2	18.2
Female	94	43.5	6	11.8	79	51.3	9	81.8
Level of education								
Low (Primary/secondary)	11	5.1	0	0	0	0	11	100
High (Tertiary)	205	94.9	51	100	154	100	0	0
Household income†								
B40 (≤MYR3440)	58	26.9	3	5.9	44	28.6	11	100
M40 (≤MYR7694)	156	72.2	46	90.2	110	71.4	0	0
T20 (≥MYR15867)	2	0.9	2	3.9	0	0	0	0
Current smoking status								
Yes	54	25	15	29.4	37	24	2	18.2
No	162	75	36	70.6	117	76	9	81.8

∗ A total of 24 participants were excluded from the study because they did not provide informed consent.

† B40, M40, and T20 represent percentages of the country's population of bottom 40%, middle 40%, and top 20%, respectively. B40: <MYR 3440 (<USD $769); M40: ≤MYR7694 (≤USD $1721); T20: ≥MYR15867 (≥USD $3549).

### 3.2 Physical performances of facemask

#### 3.2.1 Penetration

##### 3.2.1.1 Main effects

A mixed-repeated-measure analysis of variance was performed to evaluate the effect of different time points on the penetration of all three types of face masks (Table 3). A significant main effect of time was observed for the surgical mask, *F* (1, 213) = 22.04, *p* < 0.001, partial η^2^ = 0.10 (medium effect), indicating a decrease in particle penetration from 4 to 8 hours. No significant time effects were found for KF94 or cloth masks.

##### 3.2.1.2 Interaction effects

There was a significant interaction between time and occupational group for the surgical mask, *F* (2, 213) = 14.13, *p* < 0.001 (Table 3). *Post hoc* pairwise comparisons (Bonferroni-adjusted) showed that penetration decreased significantly after 8 hours among cleaners (*p* < 0.001) (Table 2).

**Table 2:** Descriptive statistics of mask penetration (%) and breathing resistance (mmH_2_O) performances based on type of mask and occupation (*N* = 216)

Type of mask	Penetration (%)∗						Breathing resistance (mmH_2_O)∗					
	Type of occupation						Type of occupation					
	Trainer		Office worker		Cleaner		Trainer		Office worker		Cleaner	
	Time in hours						Time in hours					
	4	8	4	8	4	8	4	8	4	8	4	8
KF94	0.5 (0.003)	0.5 (0.001)	0.6 (0.2)	0.6 (0.1)	0.5 (0.01)	0.5 (0.01)	12.8 (1.9)	12.9 (1.7)	12.8 (1.7)	13.1 (1.7)	15.7 (0.1)	15.6 (0.1)
Surgical	9.5 (0.02)	9.5 (0.02)	9.5 (0.4)	9.5 (0.02)	56.2 (6.1)	53.6 (7.9)	5.3 (0.3)	9.7 (0.5)	2.5 (0.3)	2.5 (0.3)	3.2 (0.6)	4.7 (0.7)
Cloth	82.6 (0.5)	82.3 (0.9)	83.5 (1.3)	83.6 (1.2)	82.3 (0.1)	82.2 (0.1)	2.5 (0.3)	2.4 (0.3)	2.5 (0.3)	2.5 (0.3)	2.5 (0.3)	2.4 (0.3)

SD, standard deviation.

∗ Mean (SD).

#### 3.2.2 Breathing resistance

##### 3.2.2.1 Main effect

Time had a significant effect on breathing resistance in surgical masks, *F* (1, 213) = 1364.18, *p* < 0.001, partial η^2^ = 0.90 (large effect), with higher resistance observed after 8 hours of wear (Table 4).

**Table 3:** Mixed-repeated-measure ANOVA results for mask penetration (%) performance based on type of mask and occupation

Type of mask	Predictor	df	Mean square	F	*p* value	Partial Ƞ^2^
KF94	Time	1	0.002	0.17	0.685	0.001
	Time∗Type of occupation	2	0.004	0.27	0.764	0.003
	Error (Time)	213	0.01			
Surgical	Time	1	27.78	22.04	<0.001∗	0.1
	Time∗Type of occupation	2	17.81	14.13	<0.001∗	0.1
	Error (Time)	213	1.26			
Cloth	Time	1	0.13	0.1	0.764	<0.001
	Time∗Type of occupation	2	1.62	1.14	0.322	0.01
	Error (Time)	213	1.42			

Note: df = degree of freedom, Ƞ^2^ = eta squared.

ANOVA, analysis of variance.

∗ significant at *p* value <0.05.

**Table 4:** Mixed-repeated-measure ANOVA results for mask breathing resistance (mmH_2_O) based on type of mask and occupation

Type of mask	Predictor	df	Mean square	F	*p*	Partial Ƞ^2^
KF94	Time	1	0.85	0.34	0.561	0.002
	Time∗Type of occupation	2	0.61	0.24	0.784	0.002
	Error (Time)	213	2.5			
Surgical	Time	1	150.29	1364.18	<0.001∗	0.9
	Time∗Type of occupation	2	188.85	1714.16	<0.001∗	0.9
	Error (Time)	213	0.1			
Cloth	Time	1	0.21	2.6	0.111	0.01
	Time∗Type of occupation	2	0.04	0.43	0.653	0.04
	Error (Time)	213	0.1			

Note: df = degree of freedom, Ƞ^2^ = eta squared.

ANOVA, analysis of variance.

∗ significant at *p* value <0.05.

##### 3.2.2.2 Interaction effect

A significant interaction between time and occupation type was also detected, *F* (2, 213) = 1714.16, *p* < 0.001 (Table 4). The *post hoc* analysis revealed significant increases in breathing resistance after 8 hours of wearing surgical mask among both trainers and cleaners (*p* < 0.001) (Table 2).

### 3.3 Physiological effects of facemask wearer

#### 3.3.1 Facial skin temperature

##### 3.3.1.1 Main effects (4-hour wear)

Significant increases in facial skin temperature were observed after 4 hours for all mask types evaluated in our study: KF94 mask, F (1, 200) = 12.39, *p* = 0.001, partial η^2^ = 0.06 (medium effect); surgical mask, F (1, 200) = 199.69, *p* < 0.001, partial η^2^ = 0.5 (large effect); and cloth mask, F (1, 200) = 10.29, *p* = 0.002, partial η^2^ = 0.05 (small effect), as shown in Table 6.

**Table 5:** Descriptive statistics of facial skin temperature and facial TEWL based on type of mask and occupation (N = 216)

Type of mask	Facial skin temperature (°C)						Facial TEWL (g/m^2^/h)					
	Type of occupation						Type of occupation					
	Trainer		Office worker		Cleaner		Trainer		Office worker		Cleaner	
	Time (4 hours)∗		Time (4 hours)∗		Time (4 hours)∗		Time (4 hours)∗		Time (4 hours)∗		Time (4 hours)∗	
	Pre	Post	Pre	Post	Pre	Post	Pre	Post	Pre	Post	Pre	Post
KF94	31.5 (0.7)	31.5 (0.8)	31.4 (0.7)	31.5 (0.7)	30.9 (0.5)	33.6 (0.5)	16.8 (3.1)	16.8 (4.1)	17 (4)	17.1 (3.6)	16.9 (2.4)	18.5 (2.2)
Surgical	30.4 (0.3)	32.3 (0.3)	31.5 (0.7)	31.7 (0.7)	30.7 (0.6)	34.3 (0.6)	17.5 (3.9)	17.3 (3.8)	16.7 (3.5)	17.3 (3.4)	17.5 (2.4)	18.9 (2.3)
Cloth	31.7 (0.7)	31.6 (0.7)	31.4 (0.8)	31.5 (0.8)	31.3 (0.6)	32.9 (0.7)	17.7 (3.3)	17.1 (3.6)	17.2 (3.9)	17.1 (3.5)	22.3 (0.5)	23.8 (0.5)

	Time (8 hours)∗		Time (8 hours)∗		Time (8 hours)∗		Time (8 hours)∗		Time (8 hours)∗		Time (8 hours)∗	
KF94	31.5 (0.7)	31.7 (0.7)	31.5 (0.6)	31.5 (0.7)	31.6 (0.7)	34.8 (0.5)	16.6 (3.6)	16.2 (3.9)	16.7 (3.7)	17 (3.4)	18.5 (2.2)	20.1 (2.1)
Surgical	30.7 (0.4)	32.8 (0.3)	31.5 (0.7)	31.6 (0.8)	31.3 (0.6)	34.5 (0.6)	17.6 (3.9)	15.7 (3.5)	16.9 (3.7)	16.9 (3.9)	17.9 (2.4)	19.5 (2.4)
Cloth	31.2 (0.8)	31.4 (0.8)	31.5 (0.7)	31.4 (0.8)	30.9 (0.5)	34.4 (0.6)	15.9 (3.3)	18.1 (3.6)	17 (3.7)	17.5 (3.5)	23.8 (0.5)	25.9 (0.4)

ANOVA, analysis of variance; SD, standard deviation; TEWL, transepidermal water loss.

∗ Mean (SD).

**Table 6:** Mixed-repeated-measure ANOVA results for facial skin temperature (°C) in KF94, surgical, and cloth masks for 4 hours and 8 hours wearing

Type of mask	Variable	Wearing duration									
		4 hours					8 hours				
		df	Mean square	F	*p value*	Partial Ƞ^2^	df	Mean square	F	*p value*	Partial Ƞ^2^
KF94	Time	1	6.59	12.39	0.001∗	0.06	1	20.68	46.73	<0.001∗	0.2
	Time∗Age	1	0.001	0.003	0.959	<0.001	1	0.12	0.26	0.611	0.001
	Time∗Gender	1	<0.001	0.001	0.979	<0.001	1	0.36	0.8	0.371	0.004
	Time∗Type of occupation	2	7.27	13.67	<0.001∗	0.12	2	16.04	36.24	<0.001∗	0.3
	Time∗Smoking	1	1.35	2.55	0.112	0.013	1	0.001	0.002	0.964	<0.001
	Error (Time)	200	0.53				200	.44			
Surgical	Time	1	70.22	199.69	<0.001∗	0.5	1	50.44	147.69	<0.001∗	0.4
	Time∗Age	1	0.15	0.42	0.519	0.002	1	0.06	0.17	0.68	0.001
	Time∗Gender	1	0.79	2.24	0.136	0.01	1	0.22	0.63	0.428	0.003
	Time∗Type of occupation	2	32.78	93.2	<0.001∗	0.5	2	34.27	100.34	<0.001∗	0.5
	Time∗Smoking	1	1.24	3.53	0.062	0.02	1	0.08	0.22	0.637	0.001
	Error (Time)	200	0.35				200	0.34			
Cloth	Time	1	6.13	10.29	0.002∗	0.05	1	18.84	37.85	<0.001∗	0.2
	Time∗Age	1	0.09	0.16	0.694	0.001	1	0.02	0.045	0.833	<0.001
	Time∗Gender	1	0.66	1.11	0.294	0.01	1	0.94	1.89	0.171	0.009
	Time∗Type of occupation	2	3.19	5.35	0.005∗	0.05	2	21.19	42.58	<0.001∗	0.3
	Time∗Smoking	1	0.1	0.17	0.681	0.001	1	0.54	1.09	0.299	0.005
	Error (Time)	200	0.59				200	0.49			

Note: df = degree of freedom, Ƞ^2^ = eta squared.

ANOVA, analysis of variance.

∗ significant at *p* value <0.05.

##### 3.3.1.2 Interaction effects (4-hour wear)

A significant interaction between mask type and occupation was found for all masks. *Post hoc* pairwise comparisons with a Bonferroni adjustment indicated a significant increase in facial skin temperature among cleaners for KF94 and cloth masks (*p* < 0.001) and across all occupation types for the surgical mask (*p* < 0.001) (Table 5).

##### 3.3.1.3 Main effects (8-hour wear)

All mask types showed further significant increases after 8 hours: KF94, F (1, 200) = 46.73, *p* < 0.001, partial η^2^ = 0.2 (large effect); surgical mask, F (1, 200) = 50.44, *p* < 0.001, partial η^2^ = 0.4 (large effect); and cloth mask, F (1, 200) = 37.85, *p* < 0.001, partial η^2^ = 0.2 (large effect), as shown in Table 6.

##### 3.3.1.4 Interaction effects (8-hour wear)

A statistically significant interaction was also observed between 8-hour mask wearing and occupation type on facial skin temperature. *Post hoc* pairwise comparisons with a Bonferroni adjustment revealed a significant difference in facial skin temperature after 8 hours of wearing KF94 and surgical masks (*p* < 0.05) among trainers and cleaners, and for the cloth mask only among cleaners (Table 5).

#### 3.3.2 Facial TEWL

##### 3.3.2.1 Main effects (4-hour wear)

The study observed no significant changes in TEWL after 4 hours of wearing KF94, surgical, or cloth masks: KF94, F (1, 200) = 0.44, *p* = 0.509, partial Ƞ2 = 0.002 (small effect); surgical, F (1, 200) = 0.21, *p* = 0.645, partial Ƞ2 = 0.001 (small effect); and cloth, F (1, 200) = 0.39, *p* = 0.535, partial Ƞ2 = 0.002 (small effect) (Table 7).

**Table 7:** Mixed-repeated-measure ANOVA results for facial TEWL (g/m^2^/h) in KF94, surgical, and cloth masks for 4 hours and 8 hours wearing

Type of mask	Variable	Wearing duration									
		4 hours					8 hours				
		df	Mean square	F	*p*	Partial Ƞ^2^	df	Mean square	F	*p*	Partial Ƞ^2^
KF94	Time	1	6.19	0.44	0.509	0.002	1	0.84	0.06	0.803	<0.001
	Time∗Age	1	8.42	0.59	0.441	0.003	1	1.71	0.13	0.722	0.001
	Time∗Gender	1	10.69	0.76	0.385	0.004	1	11.38	0.85	0.359	0.004
	Time∗Type of occupation	2	2.59	0.18	0.833	0.002	2	15.22	1.13	0.324	0.01
	Time∗Smoking	1	0.27	0.02	0.89	<0.001	1	12.54	0.93	0.335	0.005
	Error (Time)	200	14.13				200	13.44			
Surgical	Time	1	2.33	0.21	0.645	0.001	1	18.11	1.24	0.268	0.006
	Time∗Age	1	0.1	0.01	0.924	<0.001	1	0.31	0.02	0.884	<0.001
	Time∗Gender	1	23.18	2.12	0.147	0.01	1	31.54	2.15	0.144	0.01
	Time∗Type of occupation	2	16.56	1.51	0.223	0.02	2	15.14	1.03	0.358	0.01
	Time∗Smoking	1	0.04	0.004	0.952	<0.001	1	6.88	0.47	0.494	0.002
	Error (Time)	200	10.94				200	14.66			
Cloth	Time	1	4.89	0.39	0.535	0.002	1	55.18	4.58	0.034∗	0.02
	Time∗Age	1	0.08	0.01	0.938	<0.001	1	12.61	1.05	0.308	0.005
	Time∗Gender	1	2.82	0.22	0.638	0.001	1	0.78	0.07	0.799	<0.001
	Time∗Type of occupation	2	6.88	0.54	0.581	0.005	2	5.77	0.48	0.62	0.005
	Time∗Smoking	1	12.81	1.01	0.315	0.005	1	10.56	0.88	0.35	0.004
	Error (Time)	200	12.65				200	12.05			

Note: df = degree of freedom, Ƞ^2^ = eta squared.

ANOVA, analysis of variance; TEWL, transepidermal water loss.

∗ significant at *p* value <0.05.

##### 3.3.2.2 Main effects (8-hour wear)

The effects of KF94, F (1, 200) = 0.06, *p* = 0.803, partial Ƞ2 = <0.001, small effect)and surgical, F (1, 200) = 1.24, p = 0.268, partial Ƞ2 = 0.006 (small effect) masks after 8 hours of wear on facial TEWL were also not significant at a *p* value less than 0.05 (Table 7). However, the effect of the cloth mask after 8 hours of wear on facial TEWL was significant F (1, 200) = 4.58, p = 0.034, partial Ƞ2 = 0.02 (small effect).

##### 3.3.2.3 Interaction effects (8-hour wear)

There was no significant interaction between the effects of cloth mask after 8 hours of wear and type of occupation on facial TEWL [F (2, 200) = 0.48, p = 0.62] (Table 7).

## 4 Discussion

The results from our study show that there is a significant decrease in both penetration and breathing resistance performance of surgical masks after prolonged use, specifically between 4 hours and 8 hours of continuous wear, indicating a potential decline in protective efficacy over time. Our findings indicate that time significantly affects the penetration rate of surgical masks, with a large effect size. This observation was consistent across both cleaner and trainer groups. These results align with recent studies that have examined the durability and efficiency of facemask over prolonged use. For instance, research has shown that prolonged wear of masks can lead to a decrease in filtration efficiency and an increase in breathing resistance due to the accumulation of moisture and particles within the mask layers, leading to structural degradation and a compromised seal against the face [17,22,27,28]. It is also plausible that the significant differences in penetration rates observed in this study are influenced not only by mask type but also by variations in material quality across the samples tested. Surgical masks may vary in quality depending on the manufacturer, particularly in the thickness and integrity of the melt-blown filter layer [[17], [18], [19]].

The findings from this study also reveal a significant impact of prolonged face mask use on facial skin temperature, with varying effects observed across different types of masks and occupations. The 4-hour wear duration resulted in statistically significant increases in facial skin temperature for all three mask types evaluated: KF94, surgical, and cloth masks. Notably, the effect size differed among the masks, with surgical masks demonstrating the most substantial impact (partial Ƞ^2^ = 0.5, indicating a large effect), followed by KF94 masks (partial Ƞ^2^ = 0.06, indicating a medium effect), and cloth masks (partial Ƞ^2^ = 0.05, indicating a small effect). This trend underscores the differential thermal impact of various mask materials and designs on the skin, likely due to differences in breathability, fit, and insulation properties [[29], [30], [31]]. The interaction between mask type and occupation further complicates the relationship between mask use and facial skin temperature. For instance, significant differences were observed in the facial skin temperature between the KF94 and cloth masks among cleaners, and across all occupations for surgical masks, particularly after 4 hours of wear.

When the duration was prolonged to 8 hours, the effect on facial skin temperature became even more pronounced, with large effect sizes noted across all mask types. The *post hoc* analysis highlighted significant differences in temperature increases between the KF94 and surgical masks for trainers and cleaners, and between the cloth masks for cleaners. These findings suggest that occupation-specific factors such as the higher OPA levels of trainers and cleaners compared to office workers may exacerbate the thermal discomfort associated with prolonged mask use which potentially impacts comfort and compliance [6,32,33]. The significant rise in facial skin temperature associated with prolonged mask-wearing and varying OPA could contribute to discomfort and potentially lead to skin issues such as irritation or dermatitis, which have been reported in previous studies [30].

Our study also investigated the impact of KF94, surgical, and cloth masks on facial TEWL over 4-hour and 8-hour periods. The results reveal that the effects of KF94 and surgical masks were not statistically significant for both durations, with small effect sizes. Similarly, the 4-hour effect of the cloth mask on TEWL was not significant, but a significant effect was observed for the 8-hour wearing period, though the effect size remained small. Previous studies reported that KF94 and surgical masks may have minimal impact on TEWL [9,12]. The notable impact seen with cloth masks over the 8-hour period suggests that prolonged wear may lead to greater effects. The cloth masks' less breathable fabric, than that of KF94 and surgical masks, likely contributes to the increased TEWL [34]. The lack of significant interaction between cloth mask usage and occupation type suggests that the effect on TEWL is independent of occupational factors in this context. This result indicates that while mask type and duration may influence TEWL, OPA differences do not substantially modify these effects.

Several limitations should be considered. The results may not be widely applicable since recruitment and sampling occurred at a single institution. However, this institution mandated the use of face masks by all workers during the COVID-19 pandemic, and we recruited all employees through universal sampling. Our study focused on specific face masks and does not allow us to generalize our findings to all face masks sold in Malaysia, regardless of manufacturer or origin. A fit test was not performed for the KF94 masks. During the data collection period, only free-size KF94 masks were available on the market due to supply shortages in Malaysia, a situation commonly encountered during the COVID-19 pandemic. As a result, even if a fit test had been conducted, participants would not have had the option to select a mask size that best fit their facial features. This limitation reflects the real-world constraints experienced during the pandemic, where tight-fitting masks like KF94 were prioritized for frontline healthcare workers. It is also important to note that the other types of face masks used in this study, namely surgical and cloth masks, are generally considered loose-fitting and do not typically require fit testing as their design does not rely on a tight seal to be effective.

Additionally, the OPA was not objectively measured for each respondent; we relied on previous classifications [21], which may have introduced exposure misclassification bias. Although this framework is one of the few available linking occupation type to daily physical activity levels, it was developed using data from a U.S. population. As such, it may not fully reflect the occupational patterns or physiological characteristics of Asian populations. Differences in body size, metabolism, and job demands between Western and East Asian contexts could limit its direct applicability. Nevertheless, in the absence of region-specific alternatives, this classification was adopted to ensure consistent categorization and comparison.

We also acknowledge that both environmental and personal factors may have influenced the study outcomes. Ambient conditions were controlled where possible, with most data collection conducted indoors at temperatures between 23°C and 26°C and relative humidity ranging from 40% to 70%. Although outdoor conditions were less controllable, most participants with high OPA including cleaners and trainers carried out their activities indoors. Personal factors such as facial hair and cosmetic use were not controlled. While facial hair may compromise the fit of tight-fitting masks like KF94, its impact on looser-fitting masks (surgical and cloth) is likely negligible. In addition, this study focused solely on facial skin temperature and TEWL as physiological response parameters. Previous studies have included additional measurements such as respiratory rate, heart rate, oxygen saturation, and carbon dioxide levels, which may provide a more comprehensive understanding of the physiological effects associated with prolonged face mask use**.** Despite these limitations, our findings can help policymakers choose appropriate face masks for different occupational settings, reflecting real workplace conditions.

## 5 Conclusion

The study concludes that using surgical masks for up to 8 hours can significantly affect their penetration efficiency and increase breathing resistance, particularly among cleaners with higher OPA. It was also noted that after 8 hours of wear, all types of face masks led to an increase in facial skin temperature among cleaners and trainers with higher OPA, compared to office workers. However, the impact on TEWL was minimal for those wearing cloth masks. While these small effects may have limited clinical significance, the differences observed with prolonged mask use highlight the need for further research into the impact on facial skin hydration. This underscores the importance of considering both the type of mask and the wearer's occupation when recommending mask use durations, especially for those in physically demanding jobs. Additionally, the differences in performance over time suggest that protocols for mask replacement or breaks are essential to maintain optimal protection during prolonged wear. Developing occupation-specific guidelines for mask use could improve compliance and reduce the risk of adverse effects associated with prolonged mask wearing.

## CRediT authorship contribution statement

**Baderin Osman:** Writing – review & editing, Supervision, Resources, Project administration, Methodology, Funding acquisition, Formal analysis, Data curation, Conceptualization. **Muhammad Zubir Yusof:** Writing – review & editing, Writing – original draft, Methodology, Formal analysis, Conceptualization. **Haalah Mahmud:** Writing – review & editing, Resources, Methodology, Formal analysis.

## Declaration of Generative AI and AI-assisted technologies in the writing process

During the preparation of this work, the author did not use any AI tools.

## Conflicts of interest

The authors declare that they have no known competing financial interests or personal relationships that could have appeared to influence the work reported in this paper.

## References

[1] World Health Organization (2021). https://www.who.int/publications/i/item/WHO-2019-nCoV-HCW_advice-2021-1.

[2] Kobayashi L.M., Marins B.R., Costa PC. dos S., Perazzo H., Castro R. (2020). Extended use or reuse of N95 respirators during COVID-19 pandemic: an overview of national regulatory authority recommendations. Infect Control Hosp Epidemiol.

[3] Dugdale C.M., Walensky R.P. (2020). Filtration efficiency, effectiveness, and availability of N95 face masks for COVID-19 prevention. JAMA Intern Med.

[4] Liu C., Li G., He Y., Zhang Z., Ding Y. (2020).

[5] Wangsan K., Sapbamrer R., Sirikul W., Panumasvivat J., Surawattanasakul V., Assavanopakun P. (2022). Effect of N95 respirator on oxygen and carbon dioxide physiologic response: a systematic review and meta-analysis. Int J Environ Res Public Health.

[6] Litwinowicz K., Choroszy M., Ornat M., Wróbel A., Waszczuk E. (2022). Bayesian network meta-analysis of face masks’ impact on human physiology. Sci Rep.

[7] Gupta D. (2020). Living with in-mask micro-climate. Med Hypotheses.

[8] Gefen A., Ousey K. (2020). Update to device-related pressure ulcers: SECURE prevention. COVID-19, face masks and skin damage. J Wound Care.

[9] Park S., Han J., Yeon Y.M., Kang N.Y., Kim E. (2021). Effect of face mask on skin characteristics changes during the COVID-19 pandemic. Skin Res Technol.

[10] Martel T., Orgill D.P. (2020). Medical device–related pressure injuries during the COVID-19 pandemic. J Wound Ostomy Continence Nurs.

[11] Barnawi G.M., Barnawi A.M., Samarkandy S. (2021). The association of the prolonged use of personal protective equipment and face mask during COVID-19 pandemic with various dermatologic disease manifestations: a systematic review. Cureus.

[12] Montero-Vilchez T., Cuenca-Barrales C., Martinez-Lopez A., Molina-Leyva A., Arias-Santiago S. (2021). Skin adverse events related to personal protective equipment: a systematic review and meta-analysis. J Eur Acad Dermatol Venereol.

[13] Kisielinski K., Giboni P., Prescher A., Klosterhalfen B., Graessel D., Funken S., Kempski O., Hirsch O. (2021). Is a mask that covers the mouth and nose free from undesirable side effects in everyday use and free of potential hazards?. Int J Environ Res Public Health.

[14] Zhao M., Liao L., Xiao W., Yu X., Wang H., Wang Q., Lin Y.L., Kilinc-Balci F.S., Price A., Chu L., Chu M.C. (2020). Household materials selection for homemade cloth face coverings and their filtration efficiency enhancement with triboelectric charging. Nano Lett.

[15] Kwong L.H., Wilson R., Kumar S., Crider Y.S., Reyes Sanchez Y., Rempel D., Pillarisetti A. (2021). Review of the breathability and filtration efficiency of common household materials for face masks. ACS Nano.

[16] Shimasaki N., Okaue A., Morimoto M., Uchida Y., Koshiba T., Tsunoda K., Arakawa S., Shinohara K. (2020). A multifaceted evaluation on the penetration resistance of protective clothing fabrics against viral liquid drops without pressure. Biocontrol Sci.

[17] Tcharkhtchi A., Abbasnezhad N., Zarbini Seydani M., Zirak N., Farzaneh S., Shirinbayan M. (2021). An overview of filtration efficiency through the masks: mechanisms of the aerosols penetration. Bioact Mater.

[18] Beesoon S., Behary N., Perwuelz A. (2020). Universal masking during COVID-19 pandemic: can textile engineering help public health? Narrative review of the evidence. Prev Med (Baltim).

[19] Yao B., Wang Y., Ye X., Zhang F., Peng Y. (2019). Impact of structural features on dynamic breathing resistance of healthcare face mask. Sci Total Environ.

[20] Nciri N., Kim N. (2023). Infrastructure in the age of pandemics: utilizing polypropylene-based mask waste for durable and sustainable road pavements. Polymers (Basel).

[21] Steeves J.A., Tudor-Locke C., Murphy R.A., King G.A., Fitzhugh E.C., Bassett D.R., Van Domelen D., Schuna J.M., Harris T.B. (2018). Daily physical activity by occupational classification in US adults: NHANES 2005-2006. J Phys Activity Health.

[22] Li X., Ding P., Deng F., Mao Y., Zhou L., Ding C., Wang Y., Luo Y., Zhou Y., MacIntyre C.R., Tang S. (2022). Wearing time and respiratory volume affect the filtration efficiency of masks against aerosols at different sizes. Environ Technol Innov.

[23] Malaysia D. of S. (2019). testing, and marking.

[24] Htwe Y.Z.N., Mamat H., Osman B., Mahmud H. (2023). Performance comparison of single and double masks: filtration efficiencies, breathing resistance and CO2 content. Arab J Sci Eng.

[25] AFNOR Spec S76-001 (2020). S76-001 Barrier Masks–Guide Minimum Requirements, Methods Testing, Making Use [Internet].

[26] IBM Corp (2015).

[27] Wang A.-B., Zhang X., Gao L.-J., Zhang T., Xu H.-J., Bi Y.-J. (2023). A review of filtration performance of protective masks. Int J Environ Res Public Health.

[28] Law C.S.W., Lan P.S., Glover G.H. (2021). Effect of wearing a face mask on fMRI BOLD contrast. Neuroimage.

[29] Chua M.H., Cheng W., Goh S.S., Kong J., Li B., Lim J.Y.C., Mao L., Wang S., Xue K., Yang L., Ye E. (2020). Face masks in the new COVID-19 normal: materials, testing, and perspectives. Research.

[30] Elisheva R. (2020). Adverse Effects Prolonged Mask Use Among Healthc Professionals During COVID-19.

[31] Scarano A., Inchingolo F., Lorusso F. (2020). Facial skin temperature and discomfort when wearing protective face masks: thermal infrared imaging evaluation and hands moving the mask. Int J Environ Res Public Health.

[32] Engeroff T., Heinsel K., Niederer D., Nienhaus A., Groneberg D.A., Vogt L. (2024). Investigating effects of FFP2 wearing during physical activity on gas exchange, metabolism and affective state using a randomized controlled trial. Sci Rep.

[33] van Kampen V., Marek E.-M., Sucker K., Jettkant B., Kendzia B., Strauß B., Ulbrich M., Deckert A., Berresheim H., Eisenhawer C., Hoffmeyer F. (2023). Influence of face masks on the subjective impairment at different physical workloads. Sci Rep.

[34] O’Kelly E., Pirog S., Ward J., Clarkson P.J. (2020). Ability of fabric face mask materials to filter ultrafine particles at coughing velocity. BMJ Open.

