# Supplementary material for: Effect of Prolonged Use of Different Facemasks on Their Physical Performance and Physiological Impact on the Wearer
Source: Saf Health Work. 2025 Jun 30;16(3):371–7. doi: 10.1016/j.shaw.2025.06.001 (PMC12490564; doi:10.1016/j.shaw.2025.06.001)
Supplement: Multimedia component 1 [file mmc1.docx]

Appendix A

Start

Prepare the DiaCAM 2 Device

Set Up Measurement Parameters

Inspect Target Area

- Identify equipment or surface to measure

- Ensure safe distance and angle

- Avoid reflective surfaces and obstructions

Capture Thermal Images

- Aim and focus on target

- Press capture button

- Save image to device

Review and Annotate Image (if needed)

- Use on-screen tools to mark hotspots

- Check temperature readings at critical points

Transfer Data to PC or Software

- Connect via USB or SD card

- Use dedicated software (e.g., CAmReport)

Analyze and Generate Report

End

Figure 1: The flowchart of the transepidermal water loss measurement process
